# WePBAS: A Weighted Pixel-Based Adaptive Segmenter for Change Detection

**DOI:** 10.3390/s19122672

**Published:** 2019-06-13

**Authors:** Wenhui Li, Jianqi Zhang, Ying Wang

**Affiliations:** College of Computer Science and Technology, Jilin University, Changchun 130012, China; liwh@jlu.edu.cn (W.L.); jluzhangjianqi@163.com (J.Z.)

**Keywords:** change detection, weighted sample, background model update mechanism, adaptive foreground counter

## Abstract

The pixel-based adaptive segmenter (PBAS) is a classic background modeling algorithm for change detection. However, it is difficult for the PBAS method to detect foreground targets in dynamic background regions. To solve this problem, based on PBAS, a weighted pixel-based adaptive segmenter named WePBAS for change detection is proposed in this paper. WePBAS uses weighted background samples as a background model. In the PBAS method, the samples in the background model are not weighted. In the weighted background sample set, the low-weight background samples typically represent the wrong background pixels and need to be replaced. Conversely, high-weight background samples need to be preserved. According to this principle, a directional background model update mechanism is proposed to improve the segmentation performance of the foreground targets in the dynamic background regions. In addition, due to the “background diffusion” mechanism, the PBAS method often identifies small intermittent motion foreground targets as background. To solve this problem, an adaptive foreground counter was added to the WePBAS to limit the “background diffusion” mechanism. The adaptive foreground counter can automatically adjust its own parameters based on videos’ characteristics. The experiments showed that the proposed method is competitive with the state-of-the-art background modeling method for change detection.

## 1. Introduction

In many computer vision applications such as video surveillance [1], object tracking [2,3], optical motion capture [4], and anomaly identification [5], precise change detection (also referred to as foreground segmentation in some works) is a very important step. Change detection is a binary classification problem. In this problem, the algorithm needs to divide the pixels in each frame into foreground or background. In change detection, background modeling is a very common method. The general idea of a background modeling method is to construct a reliable reference model of the background, and then find the difference between the current frame and the background model. A location with significant difference can be regarded as the foreground, and the rest of the image as the background. Foreground objects are generally moving targets. However, not all moving objects are foreground objects, such as swaying trees. At the same time, not all stationary objects belong to the background, such as vehicles that are temporarily stationary because of a red traffic light.

The most important step in the background modeling method is the process of extracting the background representation in a video with various real environments. If the background is stationary, an effective background model is very easy to obtain. The single Gaussian background model [6] is the preferred method for dealing with single-modal backgrounds. But in the real world, the background often contains tiny movements. So, the background usually has multiple modes. Stauffer and Grimson et al. [7] use the Gaussian Mixture Model (GMM) to deal with multi-modal backgrounds in 1999. In the GMM, a background is represented by a set of weighted Gaussian distributions. Each Gaussian distribution can represent one modality of the background. Because the GMM [7] cannot adaptively adjust its own parameters, Zivkovic [8] later developed an improved adaptive Gaussian Mixture Model. The method in paper [8] can automatically choose the appropriate number of Gaussian distributions. The method in paper [9] can adaptively learn the learning rate parameters of the Gaussian model using a set of random samples which were recently observed. Due to the effectiveness of the GMM, many background modeling methods have been implemented based on it [10,11]. However, the algorithms based on GMM are computationally expensive because they need to calculate the mean and variance of all Gaussian distributions in each frame. The GMM-based algorithms generally reduce the number of modalities in the background model in order to reduce computational costs. But this also reduces the confidence of the background model. Furthermore, Barnich [12] found that many natural images exhibit non-Gaussian statistics feature. All of the above algorithms can be called the background modeling method based on probability distribution function (PDF).

To solve these problems, in recent years, sample-based background modeling methods have become popular. A typical feature of these methods is to directly use the current image sample as the background model. In the background update phase, these methods directly replace the samples of the background model with pixel samples. Sample-based background modeling methods do not have parameters such as mean and variance, which simplifies the calculation. At the same time, the mechanism of using pixel samples as a background model can effectively process background pixels with non-Gaussian statistical characteristics. 

The following is a brief introduction to some sample-based background modeling methods. Wang and Hanzi [13] proposed a consensus-based algorithm, referred to as SACON. In this method, the background model is defined by the *N* most recently observed pixel values. *N* is the number of the samples of the background model. In model updating, SACON replaces the oldest background model pixel value with the latest observed pixel value. This update mode is called “first in, first out” strategy. The “first in, first out” strategy ensures that the background model can be updated in time. However, there is no evidence to suggest the oldest background model is the least reliable. Later, Barnich and Olivier [12] created the ViBe method. Unlike SACON’s background update strategy, ViBe uses a random update mechanism. That means the background samples to be updated are randomly chosen. In addition, a randomly selected neighbor pixel will be “learned” into the background model (called “background diffusion” in the approach). As before, the updated background samples are randomly selected. The ViBe algorithm has achieved great success both in terms of running speed and detection performance. Based on the ViBe algorithm, the PBAS algorithm [14] and the SuBSENSE algorithm [15] were born. Both algorithms can adaptively adjust their own two key parameters: distance threshold and learning rate. But ViBe’s “random update” mechanism is not changed in the two methods. Due to the influence of the “random update” mechanism, the algorithm inevitably replaces the correct background samples with the fake ones. To alleviate this adverse effect, the PBAS algorithm and the SuBSENSE algorithm have to increase the number of background samples of the model. Subsequently, in paper [16], Jiang discussed weighted samples in the ViBe algorithm, and carried out an experiment on the SuBSENSE algorithm [15]. The experiment finally got good results. In [16], the algorithm used always updates the background model with the smallest weight when it executes a background model’s update. However, some low-weight background samples may be the background which is occluded by the temporarily stationary foreground targets. In the same year, Zhong et al. [17] introduced the mechanism of foreground counter to improve the performance of PBAS method in an intermittent object motion scene. However, in the paper [17], the behavior of using different manually adjusted parameters of the foreground counter for different videos obviously does not meet the requirement of uniform parameters. At the same time, the introduction of a foreground counter also reduces the detection effect in dynamic background scenes.

In the last few years, with the development of deep learning, methods of processing change detection with Convolutional Neural Networks (CNN) began to appear [18,19,20]. The CNN-based change detection methods generally perform better than PDF-based or sample-based background modeling methods. However, since the CNN-based change detection methods are of supervised machine learning methods, they need a lot of labeled training sample data. Besides, training CNN requires some hardware support and a lot of time. More seriously, some CNN-based change detection methods can only handle specific scenarios [18]. In contrast, PDF-based and sample-based background modeling methods do not have this problem. Due to the inflexibility of CNN-based methods, the WePBAS method is not compared with them in the experimental part.

The main contributions of this paper are summarized below:

1. The concept of weighted background samples is introduced to build a more reliable background model. Based on weighted background samples, a directional background model updating mechanism is proposed. The mechanism consists of two parts. One is a minimum weight updating strategy, which is used to remove background samples with the minimum weight in the background model. The other is a shortest matching distance updating strategy, which is used to fine-tune the background model. The mechanism can effectively improve the segmentation performance of the foreground targets in dynamic background regions.
2. An adaptive foreground counter is proposed to prevent “background diffusion” mechanism from reducing the detection performance of small intermittent moving targets. The validity of foreground counter has been fully demonstrated in [17], where the authors use different parameters that are manually set for each video. In contrast, the proposed adaptive foreground counter is able to automatically adjust the counter parameters according to videos’ characteristics.

The WePBAS is tested on the data sets CDnet2012 and CDnet2014 provided by the Change Detection Challenge website [21,22]. These data sets contain test videos of challenging scenarios in a large number of real-world. The WePBAS has shown great progress compared to the PBAS algorithm, and it is competitive with most state-of-the-art methods.

The rest of this article is organized as follows. In Section 2, the proposed method is described in detail. In Section 3, the specific values of the parameters used in the proposed method are discussed. The final experimental results of the proposed method are also presented and compared with other algorithms. In Section 4, the progress and shortcomings of the WePBAS are discussed

## 2. The Proposed Method

### 2.1. The Pixel-Based Adaptive Segmenter Method

**Figure 1:**
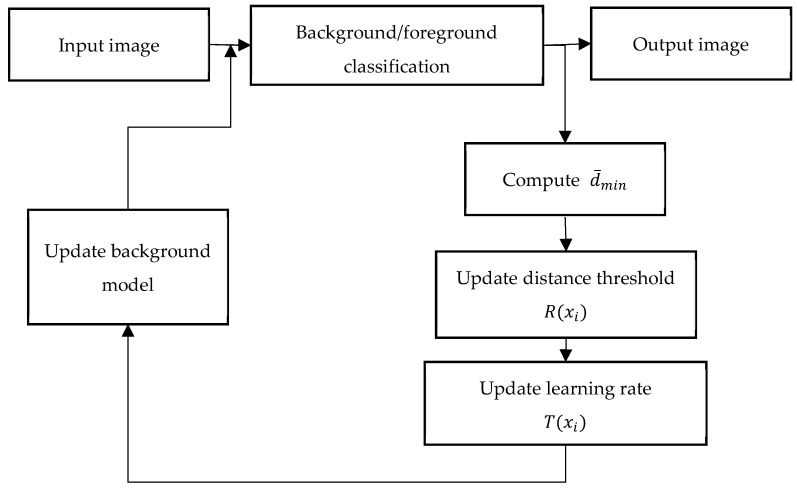
Overview of the pixel-based adaptive segmenter (PBAS) method.

The pixel-based adaptive segmenter (PBAS) [14] method is based on the ViBe [12] method which cannot adaptively adjust two important parameters: the distance threshold and the learning rate. This limits the capabilities of the ViBe method. The PBAS method changes this. Firstly, it records the value of the minimum matching distance between the pixel and its background model which is recorded as $d_{min}$. The algorithm continuously records $d_{min}$ for the latest *N* frames. Then PBAS method calculate the means of $d_{min}$, recorded as ${\bar{d}}_{min}$. Finally, the method uses ${\bar{d}}_{min}$ to update the distance threshold and the learning rate in each frame (Equations (5) and (6)). The process diagram of the PBAS algorithm is shown in Figure 1.

In the PBAS method, the background model is defined as

(1) $$B\left(x_{i}\right)=\;\{B_{1}\left(x_{i}\right),\;B_{2}\left(x_{i}\right),\;\ldots ,\;B_{N}\left(x_{i}\right)\},$$

where $x_{i}$ means the *i*-th pixel, and $B_{k}\left(x_{i}\right)$ means the pixel’s *k*-th background sample of $B\left(x_{i}\right)$. The background model contains *N* background samples to represent multiple modalities of the background. $B\left(x_{i}\right)$ is initialized by the *N* pixel values that are firstly observed by the algorithm. *N* is a fixed constant in the PBAS method. $B_{j}\left(x_{i}\right)$ consists the pixel’s value $v_{j}\left(x_{i}\right)$, gradient value $m_{j}\left(x_{i}\right)$:

(2) $$B_{j}\left(x_{i}\right)=\left\{v_{j}\left(x_{i}\right),\;m_{j}\left(x_{i}\right)\right\},\;j=1,2,\ldots ,N$$

The foreground segmentation mask is calculated as:

(3) $$F\left(x_{i}\right)=\;\{\begin{array}{l} 1,\;\#\left\{dist\left(x_{i},\;B_{k}\left(x_{i}\right)\right)<R\left(x_{i}\right)\right\}<{\#}_{min}\\ 0,\;\mathrm{else} \end{array}$$

where *F* = 1 means foreground, otherwise background. $B_{k}\left(x_{i}\right)$ denotes the *k*-th sample in the background model. #{...} denotes the number of the background samples which satisfy the condition in the brackets. $\mathrm{dist}\left(x_{i},{\;B}_{k}\left(x_{i}\right)\right)$ for each channel is calculated as follows:

(4) $$dist\left(x_{i},\;B_{k}\left(x_{i}\right)\right)=\left|v\left(x_{i}\right)-v_{j}\left(x_{i}\right)\right|+(c/{\bar{I}}_{m})*\left|m\left(x_{i}\right)-m_{j}\left(x_{i}\right)\right|$$

where *c* is a fixed parameter. ${\bar{I}}_{m}$ is the mean of the gradient values of all pixels in the previous frame. 

In Equation (3), $R\left(x_{i}\right)$ denotes $x_{i}$’s distance threshold. $R\left(x_{i}\right)$ needs to automatically adjust as follows:

(5) $$\left(x_{i}\right)=\;\{\begin{array}{ll} R\left(x_{i}\right)*\left(1-R_{inc/dec}\right), & \mathrm{if}\;R\left(x_{i}\right)>\;{\bar{d}}_{\min}*R_{scale}\\ R\left(x_{i}\right)*\left(1+R_{inc/dec}\right), & \mathrm{else} \end{array}$$

where $R_{inc/dec}$ and $R_{scale}$ are fixed parameters in PBAS. *R_lower* is the lower bound of $R\left(x_{i}\right)$. In PBAS, *R_lower* is a fixed parameter which is set to 18. The other parameter is learning rate $T\left(x_{i}\right)$. The PBAS method defines the updating rules of $T\left(x_{i}\right)$ as follows:

(6) $$T\left(x_{i}\right)=\;\{\begin{array}{l} T\left(x_{i}\right)+T_{inc}/{\bar{d}}_{min}\left(x_{i}\right),\;\mathrm{if}\;F\left(x_{i}\right)=1\\ T\left(x_{i}\right)-T_{dec}/{\bar{d}}_{min}\left(x_{i}\right),\;\mathrm{if}\;F\left(x_{i}\right)=0 \end{array}\;$$

where $T_{inc}$ and $T_{dec}$ are fixed parameters. The update speed of the background model is inversely related with $T\left(x_{i}\right)$. The range of $T\left(x_{i}\right)$’s variation is specified by the PBAS method to prevent the background model from being updated too quickly or too slowly.

### 2.2. The Proposed Method

**Figure 2:**
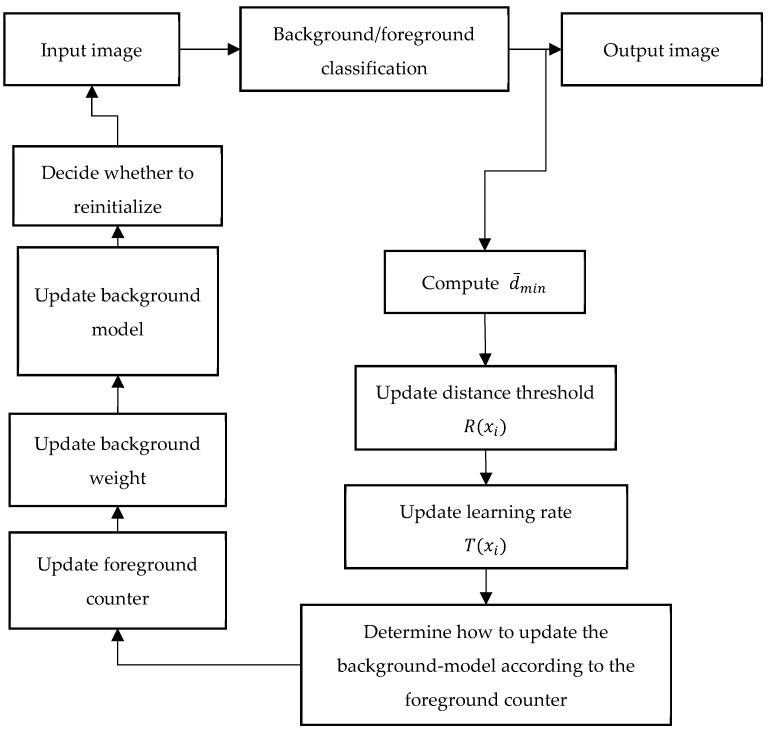
Overview of the proposed method.

The process diagram of the proposed method is shown in Figure 2. In this section, we explain in detail the similarities and differences between our WePBAS algorithm and PBAS algorithm. The segmentation decision, background model update mechanism, preprocessing, and reinitialization part of the WePBAS are introduced in this section. 

#### 2.2.1. Segmentation Decision

The goal of change detection is to obtain a binary image in which pixels are divided into foreground and background. The decision process is performed by comparing the difference between the current pixel and its background model. In our approach, the background model of the pixel $x_{i}$ is as same as PBAS’s (see Equation (1)).

A difference between our model and the PBAS algorithm is that each background sample $B_{k}\left(x_{i}\right)$ consists of three parts: (i) a background pixel value $v_{k}\left(x_{i}\right)$; (ii) a background gradient value $m_{k}\left(x_{i}\right)$; (iii) the weight $w_{k}\left(x_{i}\right)$. $B_{k}\left(x_{i}\right)$ is represented as:

(7) $$\;B_{k}\left(x_{i}\right)=\left\{v_{k}\left(x_{i}\right),\;m_{k}\left(x_{i}\right),w_{k}\left(x_{i}\right)\right\},\;k=1,2,\ldots ,N$$

where *i* is the pixel number, *k* is the background sample number.

The sum of the weights of all the samples in the background model is not normalized. Each weight has an upper limit, recorded as *max_w*, and a lower limit. The lower limit is zero. In our method, if the number of background samples matched to a pixel is greater than or equal to ${\#}_{min}$ or the sum of the weight of the matched background model is greater than or equal to $w_{b}$, the pixel will be judged as the background point, otherwise it is the foreground point. ${\#}_{min}$ is a fixed parameter which is set to 2 in our method, since this valve has been demonstrated in [12] to be capable of resisting noise. $w_{b}$ is a fixed parameter. Its value will be discussed in the experimental section. The segmentation mask is calculated as:

(8) $$F\left(x_{i}\right)=\;\{\begin{array}{l} 0,\;\#\left\{dist\left(x_{i},\;B_{k}\left(x_{i}\right)\right)<\alpha *R\left(x_{i}\right)\right\}\geq {\#}_{min}\;\mathrm{or}\;sum\left(w\right)\geq w\_b\\ 1,\;else \end{array}$$

where *i* is the pixel number, *k* is the background sample number. #{...} denotes the number of the background samples which satisfy the condition in the brackets. *F* = 1 means foreground, otherwise background. α and *w_b* is the fixed values which will be discussed in the experimental section. sum(w) is the sum of the weights of matched background samples. Unlike the PBAS algorithm which uses the distance of the three channels separately, the distance calculation formula between the pixel and the background samples of our WePBAS is described as follows:

(9) $$dist\left(x_{i},\;B_{k}\left(x_{i}\right)\right)=\;\sqrt{d_{r}{\left(x_{i},B_{j}\left(x_{i}\right)\right)}^{2}+d_{g}{\left(x_{i},B_{j}\left(x_{i}\right)\right)}^{2}+d_{b}{\left(x_{i},B_{j}\left(x_{i}\right)\right)}^{2}}$$

where $d_{r}$, $d_{g}$, and $d_{b}$ represent the calculated distances under the three channels of red, green, and blue, respectively. $B_{k}\left(x_{i}\right)$ means the *k*-th background sample of the *i*-th pixel $x_{i}$. *A* matches *B* if the following condition is met:

(10) dist(A, B)<R(A)

where *R*(*A*) is the distance threshold at pixel *A*. The distance threshold is calculated by Equation (5).

$d_{r}$, $d_{g}$, and $d_{b}$ are calculated as follows:

(11) $$\;d_{r/g/b}\left(x_{i},\;B_{k}\left(x_{i}\right)\right)=\left|v\left(x_{i}\right)-v_{k}\left(x_{i}\right)\right|+\beta *\left|m\left(x_{i}\right)-m_{k}\left(x_{i}\right)\right|$$

where $x_{i}$ is the *i*-th pixel. $v\left(x_{i}\right)$ and $m\left(x_{i}\right)$ represent the pixel value and the gradient value of $x_{i}$, respectively. $B_{k}\left(x_{i}\right)$ is the k-th background sample of $x_{i}$. $v_{k}\left(x_{i}\right)$ and $m_{k}\left(x_{i}\right)$ represent the pixel value and the gradient value of $B_{k}\left(x_{i}\right)$, respectively. β is a fixed parameter which will be discussed in the experimental section. Equation (11) is a little different from Equation (4). In the experiment, we find that the effect of ${\bar{I}}_{m}$ is slight, but the exist of ${\bar{I}}_{m}$ can increase the computational complexity of the algorithm. Thus, we replaced c/${\bar{I}}_{m}$ with a fixed constant β.

The initialization of the background model is the same as in the PBAS algorithm. The weights of the samples of the background model at initialization is set to *init_w* which is a fixed parameter. The value of *init_w* will be discussed in the experimental part.

#### 2.2.2. Background-Model Updating

The “random update” strategy inevitably causes the correct samples in the background model to be replaced by the wrong samples. So, the “random update” strategy causes pixels in the dynamic background areas to have larger distance thresholds. Larger distance thresholds make it hard for the algorithm to discriminate the foreground targets of these regions from the background. The weighted background sample and the directional background model update mechanism we designed can solve this problem. At the same time, the application of the foreground counter can suppress the “background diffusion” which make the algorithm identify small intermittent motion foreground targets as background. The validity of foreground counter has been fully demonstrated in [17]. The foreground counter of [17] needs to manually set the best parameters for different videos. Our adaptive foreground counter automatically selects the best parameters based on the videos’ characteristics.

In the proposed method, we introduce not only the concept of weighted background samples in the background model, but also the adaptive foreground counter. The proposed method is the same as the paper [17] in the usage of the foreground counter COM. The directional background model update mechanism is designed to help the algorithm select the background samples that need to be updated. The mechanism consists of two parts. One is the minimum weight updating strategy, which is used to remove the background samples with the minimum weight in the background model. The other is the shortest matching distance updating strategy, which is used to fine-tune the background model.

Before describing the background update module in detail, there are two parameters that need to be introduced in advance—*Tf* and *Tb*. *Tf* and *Tb* are the parameters of the foreground counter in the paper [17]. They also exist in the proposed algorithm. *Tf* controls the time that the algorithm starts to update the background pixels of an object. *Tb* controls the time that begins to weaken the diffusion effect of background updating for an object.

At the beginning of the algorithm, the foreground counter for each pixel is initialized to zero. The foreground counter records the times that each pixel is continuously identified as the foreground pixel.

When the pixel point is determined as the foreground point, if the value of the foreground counter COM is greater than *Tf*, the algorithm uses the information of the current pixel to replace the information of the background sample with the smallest weight among the corresponding *N* background samples. At this time, the background sample added does not match any of the previous background samples. So, the WePBAS updates the background model with minimum weight update strategy. The minimum weight update strategy ensures that invalid background samples can be replaced. The matching rule is shown in Equation (10). *Tf* is a parameter determined after the preprocessing phase of the algorithm. The updated background sample’s weight is set to *init_w*. The algorithm then updates the foreground counter COM. The update method of the foreground counter COM is the same as that in the paper [17]:

(12) COM(x)=COM(x)+1

where *x* is the pixel that the algorithm is processing. 

If the pixel is judged as the background, the pixel value and gradient value of the background sample which has the smallest matching distance to the current pixel is replaced by that of the current pixel. This update is only performed with probability *p* = 1/T(x). At this point, the newly added sample matches one or several previous background samples. So, the WePBAS updates the background model with shortest match distance update strategy. The calculation of the matching distance is shown in Equation (9). The shortest match distance update strategy allows the background model to adapt to slow changes in the background.

When the pixel point is determined as the background point and the foreground counter COM > *Tb*, the algorithm has a probability of 1/T(x) to use a randomly selected neighborhood pixel $x^{\prime }$ to update the $x^{\prime }$’s background model’s sample $B_{j}\left(x^{\prime }\right)$ whose weight is the smallest. Here, the WePBAS also uses the minimum weight update strategy. The value of the parameter *Tb* has been discussed in detail in the paper [17], where *Tb* is set to 20. The updated background samples’ weight is set to *init_w*. In paper [14], this behavior of updating the background model of the pixels around the background points is called “diffusion”.

When pixel x is judged as the background point, COM(*x*) is set to zero. In paper [17], the validity of the foreground counter COM has been fully proved

Regardless of whether the current pixel is judged to be foreground or background, the weights of the background samples are updated as follows:

(13) $$w_{k}\left(x_{i}\right)=\;\{\begin{array}{l} w_{k}\left(x_{i}\right)+delta\_w,\;\mathrm{if}\;B_{k}\left(x_{i}\right)\text{ is matched with pixel }x_{i}\\ w_{k}\left(x_{i}\right)-del\mathrm{ta}\_w,\text{ otherwise} \end{array}$$

where $x_{i}$ is the *i*-th pixel. $B_{k}\left(x_{i}\right)$ represents the *k*-th background sample of $x_{i}$. $w_{k}\left(x_{i}\right)$ represents the weight of $B_{k}\left(x_{i}\right)$. delta_w is a fixed parameter which will be discussed in the experimental section. In our method, the sum of the weights of the background samples is not normalized. The lower limit of the weight of the background sample is zero. The upper limit of the weight is *max_w* which is a fixed parameter. The value of *max_w* will be discussed in the experimental part.

**Figure 3:**
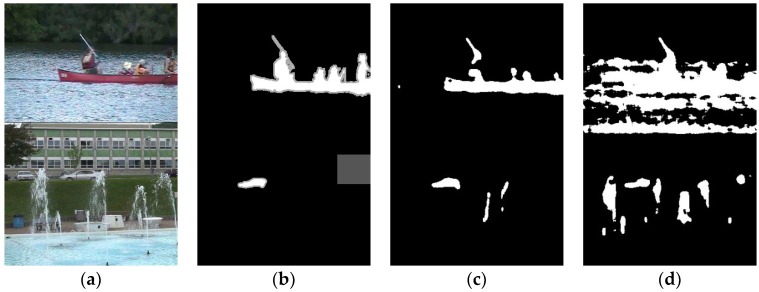
Example of the detection results of our proposed method. (**a**) Frame in the video. (**b**) Ground truth. (**c**) Results of the proposed method which uses the update mode in weighted pixel-based adaptive segmenter (WePBAS). (**d**) Results of the proposed method which uses the weight update mode in WeSamBE.

In WeSamBE [16], the weight update occurs only when the current pixel is determined to be the background point. We experiment with the above two weight update modes on the PBAS algorithm, and find that the former has a better effect on the PBAS algorithm (Figure 3).

#### 2.2.3. Algorithm Preprocessing

The algorithm preprocessing only needs to determine the value of *Tf*, which is the parameter of the foreground counter. 

In paper [17], the foreground counter has two parameters: *Tf* and *Tb*. The experiment of paper [17] has obtained the best value of *Tb* which is set to 20. However, the value of *Tf* in paper [17] is uncertain. In the experiment of the paper [17], the values of *Tf* of different videos are different. 

In our experiments, we find that the optimal value of *Tf* is closely related to the distribution of the distance threshold *R*(*x*) of all pixels of the current image. This relationship is actually easy to understand. The larger the *Tf* value, the less likely the information of the foreground pixel is to be learned by the background model, and the better the detection effect of the algorithm for the objects which are moving intermittently. The smaller the *Tf* value, the easier it is for the foreground pixel information to be learned by the background model, and the better the noise suppression of the algorithm on the dynamic background regions. So, we can draw a simple inference: the larger the proportion of the dynamic background area in the image, the smaller value of *Tf* is needed. This inference is also fully confirmed in our experiments. It should be noted that the distance threshold *R*(*x*) of the pixels of the dynamic background area is generally large to suppress the noise that often occurs.

**Figure 4:**
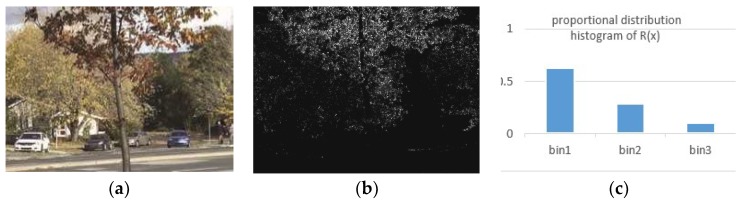
Example for the distribution of *R*(*x*). (**a**) Input image. (**b**) Distribution of *R*(*x*). The higher value of the pixel represents the larger *R*(*x*). (**c**) The proportional distribution histogram of *R*(*x*) in the input image.

In the algorithm, the histogram of the distance threshold of all pixels in an image is constructed by setting the number of bins to 3. Note distance threshold *R*(*x*)’s lower bound is *R_lower* (*R_lower* is set to 18 in PBAS method). The first bin’s *R*(*x*) is equal to *R_lower*. The second bin’s *R*(*x*) is in the range of (*R_lower*, 3*R_lower] The third bin’s *R*(*x*) is in the range of (3*R_lower, +∞). There is an example of the proportional distribution histogram of *R*(*x*) of an image (Figure 4). 

We can get a percentage distribution histogram from each input image. The normalized histogram can be written as a ternary vector form:


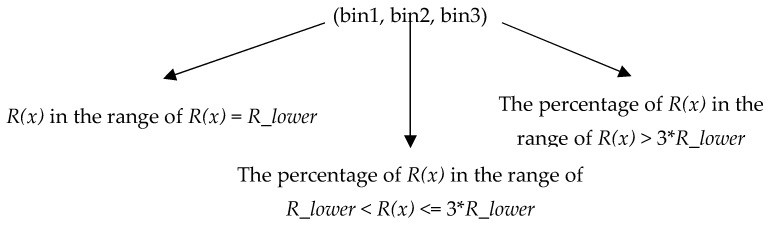


In the experiment, we find that if the background does not change drastically, the data of this histogram is basically no longer changed after the algorithm has processed 300 frames. Therefore, when the algorithm reaches the 300th frame, the optimal *Tf* value of the algorithm will be determined according to the histogram of the distance threshold. When the algorithm processes the first 300 frames, because the algorithm cannot determine the optimal *Tf* value, we set the *Tf* to 200 at this stage. This stage is called the algorithm preprocessing stage. How to get the best value of *Tf* according to the ternary vector will be discussed in the experimental part.

#### 2.2.4. Model Reinitialization

In actual scenes, the lighting may suddenly change. This situation can lead to the collapse of the background model. So, the algorithm must identify this situation and quickly update the background model to fit this situation. 

A frame-level analysis model similar to the one in the paper [23] is added to the proposed method. In paper [23], the authors believe that when the percentage of pixels (*disp*) that are significantly different between the current video image and the background image exceeds 50%, the illumination can be considered to have changed. For the sake of simplicity, the proposed method directly treats the percentage of foreground pixels as *disp*. 

From the experiment we observed that the illumination change does not significantly affect the optimal value of *Tf*. Intuitively, this is easy to understand: the lighting change does not make the static background become the dynamic background. So, after detecting the illumination change, the proposed algorithm does not recalculate the optimal value of *Tf*.

When the algorithm detects a change in illumination, the algorithm will set *Tf* to 30 in the next 100 frames to quickly update the background model. After 100 frames, *Tf* reverts to its original value.

The proposed method is summarized in Algorithm 1.

**Algorithm 1:** A Robust Background Modeling Updating Algorithm.**Input:** A frame**Output:** A binary image**Initialization:** First *N* frames are used to initialize the *N* samples of the background model. Foreground counter COM is set to 0. Weight w(x) is set to *init_w*. Learning rate *T*(*x*) and distance threshold *R*(*x*) is initialized to 18, just like in the PBAS method. The algorithm sets *Tf* to 200 when processing the first 300 frames. After the algorithm has processed 300 frames, the value of *Tf* will be determined by the distribution histogram of the distance threshold *R*(*x*) at the 300th frame.
**Procedure:**
1. Pixel *x* is classified as a foreground pixel or background pixel;2. If *x* is classified as a background pixel (a)There is a 1/*T*(*x*) probability of this happening that the algorithm uses *x* to update the background sample *B*(*x*) whose matching distance to x is the smallest;(b)If COM(x) > *Tb*, randomly select the *x*’s neighboring pixel p and use pixel p to update background sample B(p) whose weight is the smallest;(c)Counter COM(*x*) is set to 0;3. If pixel x is classified as a foreground pixel(a)Update COM(*x*) using Equation (12);(b)If COM(x) > *Tf*, update the background sample *B*(*x*) whose weight is the smallest one;4. Update *R*(*x*) and *T*(*x*) using Equation (5) and Equation (6);5. Update each background sample’s weight using Equation (13).

## 3. Experimental Results and Analysis

In this section, the performance of the proposed method is shown. Firstly, the common evaluation criteria and the benchmark test datasets on which we evaluate the proposed method are introduced. Secondly, the values of the various parameters of the proposed method are discussed. Finally, the results of the proposed method are compared with that of other algorithms on the benchmark data sets. 

### 3.1. Test Dataset and Evaluation Metrics

The two test datasets used in the experiment are Change Detection Challenge 2012 (CDnet2012) [21] and Change Detection Challenge 2014 (CDnet2014) [11]. The CDnet2012 dataset has 31 videos of six categories including baseline, dynamic background, camera jitter, shadow, intermittent object motion, and thermal. The CDnet2014 dataset is an expanded version of the dataset CDnet2012. In addition to all six categories of videos in CDnet2012, CDnet2014 supplements 22 videos in five categories: bad weather, low frame-rate, night video, PTZ (Pan/Tilt/Zoom), and turbulence. The metrics used to quantify the detection results are as follows:

(1) Recall (Re):TP/(TP + FN)
(2) Specificity (SP):TN/(TN+FP)
(3) False Positive Rate (FPR): FP/(FP + TN)
(4) False Negative Rate (FNR): FN/(TP + FN)
(5) Percentage of Wrong Classifications (PWC):100 * (FN + FP)/(TP +FN +FP +TN)
(6) F-Measure:2*Pr * Re/(Pr + Re)
(7) Precision (Pr): TP/(TP + FP)

Here, TP is the number of correctly detected foreground pixels. TN is the number of correctly detected background pixels. FP is the number of background pixels that are incorrectly marked as foreground pixels. And FN is the number of foreground pixels that are incorrectly marked as background pixels.

### 3.2. Tf Setting in Algorithm Preprocessing

At the end of the Section 2, we introduce the algorithmic preprocessing of the proposed method. The algorithm preprocessing selects the most appropriate value of *Tf* based on the distance threshold histogram of the 300-th image of the videos. The distance threshold histogram can be written as a ternary vector: (bin1, bin2, bin3). We first use the clustering algorithm to divide all videos of the CDnet2012 dataset into four categories based on the most appropriate *Tf* value of the videos. The specific division results are as follows:

(a) Fully static background videos: office, PETS2006, corridor, diningRoom, lakeSide, library.
(b) Static background videos: sofa, abandonedBox, parking, streetLight, tramstop, pedestrians, bungalows, copyMachine, cubicle.
(c) General videos: fountain01, fountain02, canoe, boats, overpass, winterDriveway, highway, badminton, boulevard, sidewalk, traffic, backdoor, busStation, peopleInShade, park.
(d) Dynamic background videos: fall.

**Figure 5:**
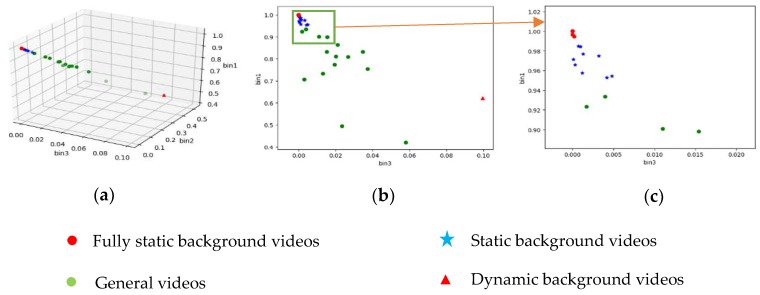
(**a**) The distribution of (bin1, bin2, bin3) of videos in CDnet2012. (**b**) The distribution of (bin1, bin3) of videos in CDnet2012. (**c**) Part of the distribution of (bin1, bin3) of videos in CDnet2012.

After experimentally measuring the histograms of *R*(*x*) of all the videos of CDnet2012 at the 300th frame, we analyzed the relationship between the histogram and the optimal value of *Tf* by plotting. We find that bin1 and bin3 are very helpful for dividing video categories (Figure 5).

According to the principle of maximum separation, the videos can be divided like this:

(a) Fully static background videos: bin1 ≥ 0.99
(b) Static background videos: 0.94 ≤ bin1 < 0.99
(c) General videos: bin1 < 0.94, and bin3 ≤ 0.08
(d) Dynamic background videos: bin3 > 0.08

**Figure 6:**
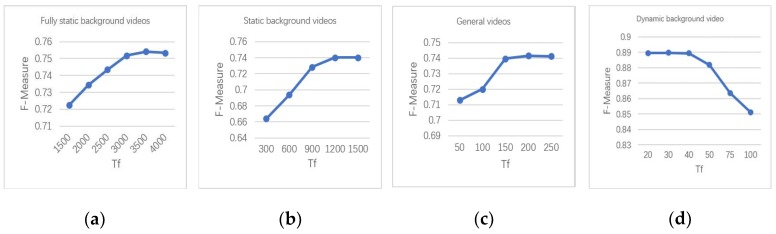
FM performance of the proposed method on different categories of videos of the CDnet2012 data set with changing *Tf* setting. (**a**) Fully static background videos. (**b**) Static background videos. (**c**) General videos. (**d**) Dynamic background videos.

The bin2 is not used as a basis for classification because, according to Figure 6, bin2 is not a good feature to distinguish videos. 

The above division criteria are used to divide the 22 videos of CDnet2014 dataset into four categories, and the results is:

(a) Fully static background videos: tunnelExit, tunnelExit_0_35fps
(b) Static background videos: blizzard, tramCrossroad_1fps, turnpike_0_5fps, fluidHighway, streetCornerAtNight, winterStreet, intermittentPan.
(c) General videos: skating, wetSnow, port_0_17fps, bridgeEntry, busyBoulvard, tramStation, continuousPan, twoPositionPTZCam, turbulence0, turbulence1, turbulence2, turbulence3.
(d) Dynamic background videos: zoomInZoomOut.

The partitioning criteria performs well on CDnet2014, except on the PTZ dataset. This is because the camera is moving in the PTZ data set. However, the basic assumption of the background modeling method is that the camera is basically in a stationary state. For each type of video, the best value of *Tf* is tested on the CDnet2012 dataset (Figure 6).

According to the experimental results on CDnet2012, the values of *Tf* of the four types of videos are set as follows:

(a) Fully static background videos: *Tf* = 3500.
(b) Static background videos: *Tf* = 1200.
(c) General videos: *Tf* = 200.
(d) Dynamic background videos: *Tf* = 30.

### 3.3. Other Parameter Settings

**Figure 7:**
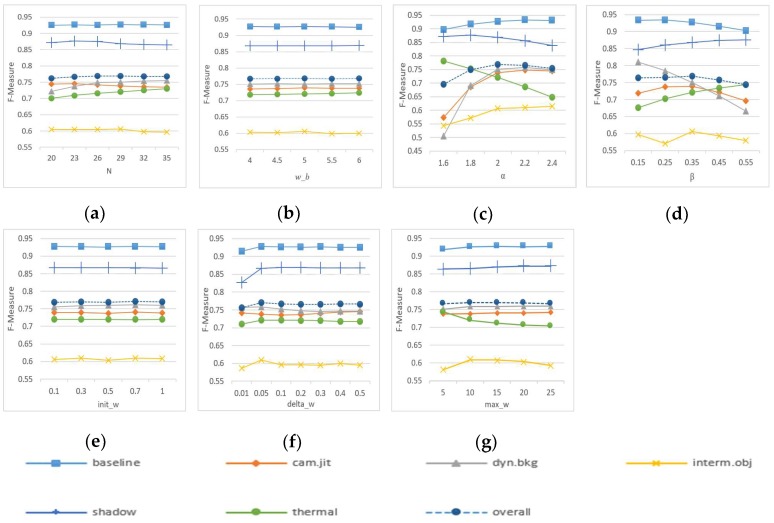
FM performance of the proposed method on different categories of videos of the CDnet2012 data set with changing parameter setting. (**a**) *N* = 29: *N* is the number of samples in the background model. In PBAS method, the background samples’ number is larger to suppress noise generated by random updates. (**b**) *w_b* = 5: *w_b* is the parameter in Equation (8) which is used to distinguish the foreground and background. (**c**) *α*= 2: *α* is the parameter in Equation (8). (**d**) *β*= 0.35: *β* is the parameter in Equation (11). (**e**) *init_w* = 0.7: *init_w* is the initial value of the weight of the background sample. (**f**) *delta_w* = 0.05: *delta_w* is the increment when the weights are updated. (**g**) *max_w* = 10: *max_w* is the upper limit of the weights.

In this section, the values of other key parameters are discussed. First of all, except for the number of samples in the background model, the values of the parameters that exist in both WePBAS method and PBAS method [14] are the same. The parameters are tested on CDnet2012 [21] dataset (Figure 7).

### 3.4. Experimental Results on CDnet2012 and CDnet2014

In Table 1, the test results of WePBAS on the CDnet2012 [21] and CDnet2014 [22] are shown. On the CDnet2012 dataset, we present the comparison of the WePBAS, PBAS [14], Zhong2017 [17], GMM-Zivkovic [8], GMM-Stauffer and Grimson [7], ViBe [12], and CDPS [24] (see Table 2). The experimental data of the comparison is derived from the results of the original paper or the authors’ online publication. As can be seen from Table 2, the proposed method has the highest FM in most scenarios of CDnet2012. And the average FM of the WePBAS in all scenes of CDnet2012 is the highest.

On the CDnet2014 dataset, we present the comparison of WePBAS, PBAS [14], GMM-Zivkovic [8], GMM-Stauffer and Grimson [7], ViBe [12], SBBS [25], and Zhong2017 [17] (see Table 3). Since the ViBe method and the PBAS method have not published the experimental results on CDnet2014, we use the test results of the two methods on the CDnet2014 dataset which can be got in the paper [17] and the paper [26]. Other results are derived from the original paper or the results published by the authors on the web. As can be seen from Table 3, the FM of the proposed method on the CDnet 2014 data set is the highest. Although the precision of WePBAS is 1.42% lower than that of SBBS [25], the recall of WePBAS is 7.14% higher than that of SBBS.

We compared the PBAS and our WePBAS method with some better methods (see Table 4). In Table 4, the speed refers to the speed when the algorithm is processing 320 × 240 images. Algorithm speed test is performed on the third generation Intel i5 CPU. Because the WePBAS algorithm needs to process additional weight information, its processing speed is slower than the PBAS algorithm. Due to the limitations of the PBAS method, our WePBAS did not achieve higher F-Measure values.

**Figure 8:**
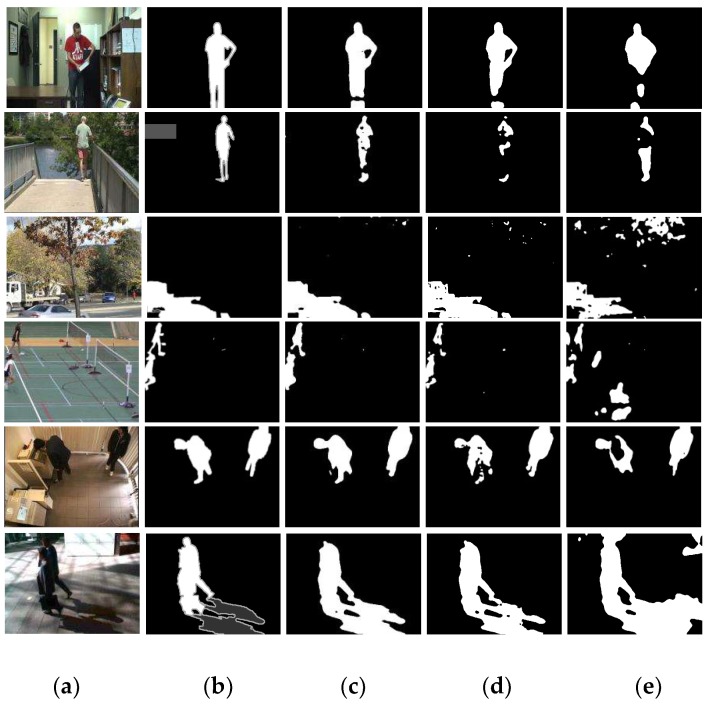
Example of foreground-segmentation results from the videos of CDnet2012 dataset. (**a**) Input video frame, (**b**) ground truth, (**c**) our proposed method, (**d**) PBAS [14], and (**e**) CDPS [24].

An example of the comparison results of foreground segmentation between different algorithms is shown in Figure 8.

The method proposed in this paper uses 9 × 9 median filtering as post-processing just like PBAS method. When processing video of different resolutions, our method firstly resizes the videos to 320 × 240 then detects the foreground. After the algorithm obtains the foreground detection binary image whose resolution is 320 × 240, the detected image is restored to the original resolution by the nearest neighbor resize.

## 4. Discussion

In this paper, based on PBAS, we propose a weight-pixel-based adaptive segmenter method named WePBAS for change detection. One of our innovations is to introduce the concept of weighted background samples for PBAS method, and design a reasonable weight update mechanism according to the characteristics of PBAS. The proposed method updates the background model by minimum weight update strategy and shortest match distance update strategy. In addition, we introduce the mechanism of the foreground counter and make the algorithm adaptively adjust the relevant parameters of the foreground counter according to the videos’ characteristics. The application of the foreground counter in background model update phase can improve the detection performance in intermittent motion scenarios. Our approach performed much better on the CDnet2012 and CDnet2014 datasets than the PBAS algorithm. On the dynamic background dataset, our algorithm achieves an improvement of f-measure close to 10% compared with the PBAS method. On the intermittent motion dataset, our algorithm achieves an improvement of f-measure close to 3% compared with the PBAS method. Our method does not run faster than the PBAS method.

## Figures and Tables

**Table 1:** The evaluation results of WePBAS on the CDnet2012 and CDnet2014.

Category	Re	Sp	FPR	FNR	PWC	F-Measure	Pr
Baseline	0.975	0.9966	0.0034	0.025	0.4211	0.9271	0.8853
Camera jitter	0.8577	0.9778	0.0221	0.1423	2.6650	0.7495	0.6782
Dynamic background	0.8255	0.9986	0.0014	0.1745	0.3623	0.7808	0.7791
Intermittent object motion	0.7195	0.9483	0.0517	0.2805	5.9417	0.6058	0.5915
Shadow	0.8735	0.9935	0.0065	0.1265	1.1971	0.8684	0.8703
Thermal	0.6351	0.9966	0.0034	0.3649	1.9896	0.721	0.9059
Bad weather	0.7201	0.9987	0.0013	0.2799	0.6414	0.7994	0.9062
Low framerate	0.8227	0.9896	0.0104	0.1773	1.2468	0.6918	0.6181
Night video	0.6741	0.9704	0.0296	0.3259	3.7228	0.4452	0.3985
PTZ(Pan/Tilt/Zoom)	0.7274	0.8960	0.1041	0.2726	10.55	0.2340	0.1812
Turbulence	0.7354	0.9999	0.0001	0.2646	0.1888	0.8235	0.9722
Average (2012)	0.8144	0.9852	0.0148	0.1856	2.0961	0.7704	0.7851
Average (2014)	0.7787	0.9787	0.0213	0.2213	2.6296	0.6924	0.7079

**Table 2:** Comparison of our WePBAS to several state-of-art methods on CDnet2012.

Algorithm	Our WePBAS	PBAS [14]	GMM-Zivkovic [8]	GMM-Stauffer and Grimson [7]	ViBe [12]	Zhong2017 [17]	CDPS [24]
$FM_{overall}$	**0.7704**	0.7532	0.6596	0.6624	0.668	0.6447	0.7281
$FM_{baseline}$	**0.9271**	0.9242	0.8382	0.8245	0.870	0.8743	0.9208
$FM_{cam.jit}$	**0.7495**	0.7220	0.5670	0.5969	0.600	0.4935	0.4865
$FM_{dyn.bkg}$	**0.7808**	0.6829	0.6328	0.6330	0.565	0.3007	0.7495
$FM_{interm.obj}$	0.6058	0.5745	0.5325	0.5207	0.507	**0.8244**	0.7406
$FM_{shadow}$	**0.8684**	0.8597	0.7319	0.7370	0.803	0.6489	0.8092
$FM_{thermal}$	0.7210	**0.7556**	0.6548	0.6621	0.665	0.7266	0.6619

**Table 3:** Comparison of our WePBAS to several state-of-art methods on CDnet2014. GMM: Gaussian Mixture Mode.

Algorithm	Our WePBAS	PBAS [14]	ViBe [12]	GMM-Zivkovic [8]	GMM-Stauffer and Grimson [7]	SBBS [25]	Zhong2017 [17]
Recall	**0.7787**	0.6397	0.3072	0.6604	0.6846	0.7073	0.7603
Precision	0.7079	0.4559	0.6322	0.5973	0.6025	**0.7221**	0.5161
FM	**0.6924**	0.5323	0.4134	0.5566	0.5707	0.6711	0.6148

**Table 4:** Comparison of the PBAS and our WePBAS to several better methods on CDnet2012.

Algorithm	Our WePBAS	PBAS [14]	SuBSENSE [15]	WeSamBE [16]	$W^{4}CD$ [23]
FM	0.7704	0.7532	0.8260	0.8197	0.5707
Speed (FPS)	7	41	45	2	4.8
